# “Natural infections” with *Trypanosoma cruzi* via the skin of mice: size of mouthparts of vectors and numbers of invading parasites

**DOI:** 10.1007/s00436-022-07516-5

**Published:** 2022-05-04

**Authors:** Barbara Waldeck, Günter A. Schaub

**Affiliations:** Zoology-Parasitology, Ruhr University, 44780 Bochum, Germany

**Keywords:** Metacyclic trypomastigotes, Mouthparts, “Natural infection”, Triatomines, *Trypanosoma cruzi*, Vector

## Abstract

Investigating parameters influencing natural infections with *Trypanosoma cruzi* via the skin, the diameters of mouthparts of different stages of triatomines vectors were measured to determine the size of the channel accessible for *T*. *cruzi* during cutaneous infection. The mean diameters of the skin-penetrating mandibles of first to fifth instar nymphs of the vector *Triatoma infestans* increased from 18 to 65 µm. The mean diameter in fourth instar nymphs of *Dipetalogaster maxima* was 86 µm. Different numbers of isolated vector-derived metacyclic trypomastigotes (10–10,000) were injected intradermally into mice. Prepatent periods, parasitemia and mortality rates were compared with those of mice obtaining 10,000 metacyclic trypomastigotes that are usually present in the first drop of faeces onto the feeding wounds of fifth and fourth instar nymphs of *T. infestans* and *D. maxima*, respectively. After injection of 50–10,000 T. *cruzi*, in all 42 mice the infection developed. An injection of 10 parasites induced an infection in 8 out of 15 mice. With increasing doses of parasites, prepatent periods tended to decrease. The level of parasitemia was higher after injection of the lowest dose. Except for one mouse all infected mice died. After placement of 10,000 metacyclic trypomastigotes onto the feeding wound of fifth or fourth instar nymphs of *T. infestans* and *D. maxima*, respectively, the infection rates of the groups, prepatent periods and the levels of parasitemia of *T*. *cruzi* in mice indicated that about 10–1,000 metacyclic trypomastigotes entered the skin via this route. For the first time, the present data emphasise the risk of an infection by infectious excreta of triatomines deposited near the feeding wound and the low number of invading parasites.

**Supplementary Information:**

The online version contains supplementary material available at 10.1007/s00436-022-07516-5.

## Introduction

Triatomines are the biggest blood-sucking insects and are predominantly found between the Great Lakes of North America and Argentina (Lent and Wygodzinsky 1979). The majority of the approximately 150 species lives in the wild, as does the biggest species, *Dipetalogaster maxima* (Uhler 1894) (Lent and Wygodzinsky 1979; Galvão 2021). Other species live peridomestically and feed on domestic animals, e.g. hens and guinea pigs, but also invade houses (Balczun et al. 2012a; Schaub et al. 2016). Only some species are strongly adapted to houses, e.g. *Triatoma infestans* (Klug 1834), *Rhodnius prolixus* Stål 1859, *Panstrongylus megistus* (Burmeister 1835) and *Triatoma dimidiata* (Latreille 1811) (Balczun et al. 2012a; Schaub et al. 2016).

Especially during the night, triatomines approach the hosts, attracted by exhaled carbon dioxide, skin odour and temperature (Lazzari et al. 2013). Then, they swing the proboscis forward and push its tip onto the skin. The proboscis covers the thin mouthparts, the mandibles and maxillae. Teeth at the tip of the mandibles cut the skin, into which only the flexible, specifically interlocked maxillae invade, swinging in the skin by joint-free bendings, until they tap a blood capillary (Geigy and Kraus 1952; Kraus 1957; Lavoipierre et al. 1959; Wirtz 1987; Tull et al. 2020). In adults of *D. maxima*, the right maxilla is 12 mm long. The left one differs in structure of the tip and is usually slightly shorter (Wirtz 1987; Tull et al. 2020). Depending on the size of the blood capillary, triatomines need about 20 min to ingest 6–12 times their own body weight, e.g. up to 3.8 ml by females of *D. maxima* (Stadler et al. 2011; Balczun et al. 2012b). Blood ingestion of the triatomine strongly increases the rate of diuresis of the Malpighian tubules which end at the border of midgut and hindgut (Maddrell 1969). The urine flows into the rectum and thereby the rectal contents are excreted, followed by drops of clear urine (Kollien and Schaub 2000). Faeces and urine are often deposited during or shortly after feeding (reviewed by Loza-Murguía and Noireau 2010).

All species of triatomines are potential vectors of *Trypanosoma cruzi* (Chagas 1909) (Jurberg and Galvão 2006) which are the causative agent of Chagas disease. According to recent estimations, approximately 6 to 7 million people are infected worldwide, mostly in Latin America (WHO 2021). The biological characteristics of *T. cruzi* strains vary considerably, some strains developing high parasitemias in mice and killing them within 3 weeks, and others showing very low, often undetectable levels of blood trypomastigotes and inducing no deaths (reviewed by Schaub et al. 2012).

The parasite is transmitted to humans via different routes, e.g. by infectious blood, organs and food as well as by the blood-sucking triatomines (Bern et al. 2020). In contrast to other tropical vector-borne diseases, *T. cruzi* is not transmitted by infectious saliva of the insect but via the faeces and urine of the vectors (Schaub et al. 2016), since the infectious metacyclic trypomastigotes develop almost exclusively in the rectum of *T. cruzi*–infected triatomines (Kollien and Schaub 2000). There, the population of *T. cruzi* contains epimastigotes and spheromastigotes, both developing in five different pathways to metacyclic trypomastigotes (summarised by Schaub et al. 2016). The surface coat is changed during metacyclogenesis, enabling an evasion of the final metacyclic trypomastigotes to the complement-mediated lysis in the mammalian host (Schaub 1991). If the excreta are deposited on mucous membranes, e.g. of lips or eyes, the metacyclic trypomastigotes can easily invade the mammalian host but they are not able to invade the host’s intact skin (Eickhoff et al. 2013). This infection route is only possible if little wounds, scratches or the feeding puncture of the insect vector enable an invasion (summarised by Schuster and Schaub 2000).

Infections via the feeding wound of the triatomine vector have been considered several times (e.g. Soares and Marsden 1986; Schuster and Schaub 2000; Eickhoff et al. 2013), but the size of wounds caused by the mouthparts of triatomines and the number of parasites invading via the feeding wound have not been determined. In the present investigation, the sizes of the mouthparts of different stages of triatomines were measured. In addition, mice were infected by intradermal injection of different doses of the metacyclic trypomastigotes or by placing metacyclic trypomastigotes of *T*. *cruzi* onto the feeding wound of a vector. A comparison of prepatent periods, parasitemias and survival time determined the number of parasites that invade the mammalian host via the feeding wound causing a patent infection.

## Materials and methods

The *T. cruzi* strain “Chile 5”, originating from Cachiyuyo in Chile, develops high parasitemias in mice and belongs to zymodeme1 (Ebert and Schaub 1983; Schaub and Schottelius 1984), according to recent multilocus sequence typing classifications to the discrete typing unit TcI (Zingales et al. 2012; Cosentino and Agüero 2012). It was cyclically passaged between mice and triatomines or stored frozen at − 78 °C (Schaub 1988). To obtain metacyclic trypomastigotes, first instar nymphs *T. infestans* were fed on *T. cruzi*–infected mice and in the following instars on hens. After feeding of 150–200 fifth instar nymphs, infectious faeces and urine were collected and metacyclic trypomastigotes carefully isolated by DEAE-Sephacel column chromatography (Schaub 1991). Since we planned to use low numbers of parasites, transition stages with an incomplete surface coat were killed by an incubation of the samples with the same volume of serum of C57 Bl/6 mice (Schaub 1991). According to counts in Neubauer chambers, this reduced the number of isolated parasites by about 10%. Infections with freshly isolated metacyclic trypomastigotes were compared in each of the four series of experiments, each one using a new isolation.

The *T. infestans* strain originated from the same village as the *T. cruzi* strain (Böker and Schaub 1984; Kollien and Schaub 1998). *D. maxima*, of which field populations only occur in the Mexican fog desert on the Baja California Peninsula in Mexico (Marsden et al. 1979), was obtained from Prof. Dr. H. Mühlpfordt, Bernhard-Nocht-Institute for Naval and Tropical Diseases, Hamburg, Germany. The triatomines were reared at 26 ± 1 °C, 60–70% relative humidity, with a 18-/6-h light/dark cycle and regularly fed on hens (Schaub 1989).

Different instars of *T. infestans* and fourth instar nymphs of *D. maxima* were allowed to ingest a small amount of blood and were then killed rapidly by cutting off the proboscis or head. The total lengths of the protruded maxillae were microscopically measured in skin biopsies of the respective region. The protruded mouthparts of 4–6 nymphs of each instar were embedded on slides in Entellan® (Merck, Darmstadt, Germany) to measure microscopically the maximal outer diameter of the mandibles.

The immunodeficient Balb/c nu/nu (nude) mice originated from the Max Planck Institute of Immunobiology, Freiburg, Germany, and the immunocompetent C57 Bl/6 mice were bread at our institute. Nude mice were chosen, since nude rats showed a good dose-dependent development of parasitemias (Schaub et al. 2001). Commercial rodent diet and water, sterilised in case of the nude mice, were available ad libitum.

All procedures with mice were conducted under deep anaesthesia and animals were carefully monitored during recovery. They were anaesthetised in all experiments using a mixture of Rompun® (Bayer, Leverkusen, Germany), Ketavet® (Upjohn, Kalamazoo, Michigan) and physiological saline (3:6:18). The C57 Bl/6 mice were shaved on their backs, using an electric razor. Since *T. cruzi* can invade the skin through minute wounds caused by shaving (Marsden 1967), this was done 24 h before infection (Eickhoff et al. 2013). Thereby, little scratches could close. Groups of four to six young females of about 10 weeks old were used in four series of experiments. For intradermal infections, a small fold of the back skin was lifted with a curved clamp and 20 µl parasite suspension containing 10, 50, 100, 1,000 and 10,000 metacyclic trypomastigotes/mouse was injected intradermally using a 25-µl Hamilton syringe (Hamilton, Darmstadt, Germany) and a 30-gauge needle (Microlance®, Becton Dickinson, Heidelberg, Germany). This volume of a solution can be correctly injected intradermally without leaking into the subcutaneous tissue (Crowle 1976). Although the mouthparts penetrate the epidermis and dermis, we chose the much more difficult intradermal injections since we suggested a rapid change of the wound channel after rejection of the mouthparts. Five different doses were not always used within each series since not enough female mice were available or other variables were tested (effects of saliva and the isolation procedure; data not included).

To obtain a natural infection route via the feeding wound of a triatomine, mice were anaesthetised and exposed to fifth instar nymphs of *T. infestans*. Since fifth instars of *T. infestans* sometimes probed more than once, we mainly used the more aggressive *D. maxima* of which fourth instar nymphs are of a similar size to fifth instars of *T. infestans* (Stadler et al. 2011). After ingestion of a small amount of blood, the triatomines were removed. The feeding site was marked with a fine pen and the mice were transferred into a moist chamber. There 10 µl parasite suspension containing 10,000 metacyclic trypomastigotes was placed onto the feeding wound. This dose is consistent with data obtained from this parasite/vector system, in which fifth instar nymphs of *T. infestans* deposited after blood ingestion a volume of 1–25 µl (mean 10 µl) in the first drop of faeces, containing 1–32,000 (mean 7,000) metacyclic trypomastigotes (Schaub and Lösch 1988). After 15 min, the suspension was removed using a smooth disposable cellulose sheet. Finally, the mice were transferred onto a heating plate to be kept warm until waking up. Since anaesthesia lasted for more than half an hour, oral infection by licking the skin at the application area was excluded.

Starting 12 (Balb/C nu/nu) or 16 (C57 Bl/6) days post infection, parasitemia was determined every day (Balb/C nu/nu) or every 2 days (C57 Bl/6) by examination of 100 microscopic fields (magnification 400 ×) of fresh blood preparations (Brener 1972). If one or more flagellates per microscopic field were counted, blood was diluted in physiological saline to determine the concentration in a special Neubauer chamber (height 0.02 mm). A conversion key was determined from both sets of data to compare all data in one standard. All mice, in which no blood trypomastigotes could be detected, were examined by xenodiagnosis (Meiser and Schaub 2011).

Pairwise statistic comparisons of prepatent periods and periods of survival were carried out using Student’s *t*-test (Statistica 9.0, Statsoft Europe, Hamburg, Germany) followed by a correlation analyses of both mean periods to the log10 of the dose. Parasitemias differed strongly within a group, allowing no statistics.

## Results

**Fig. 1:**
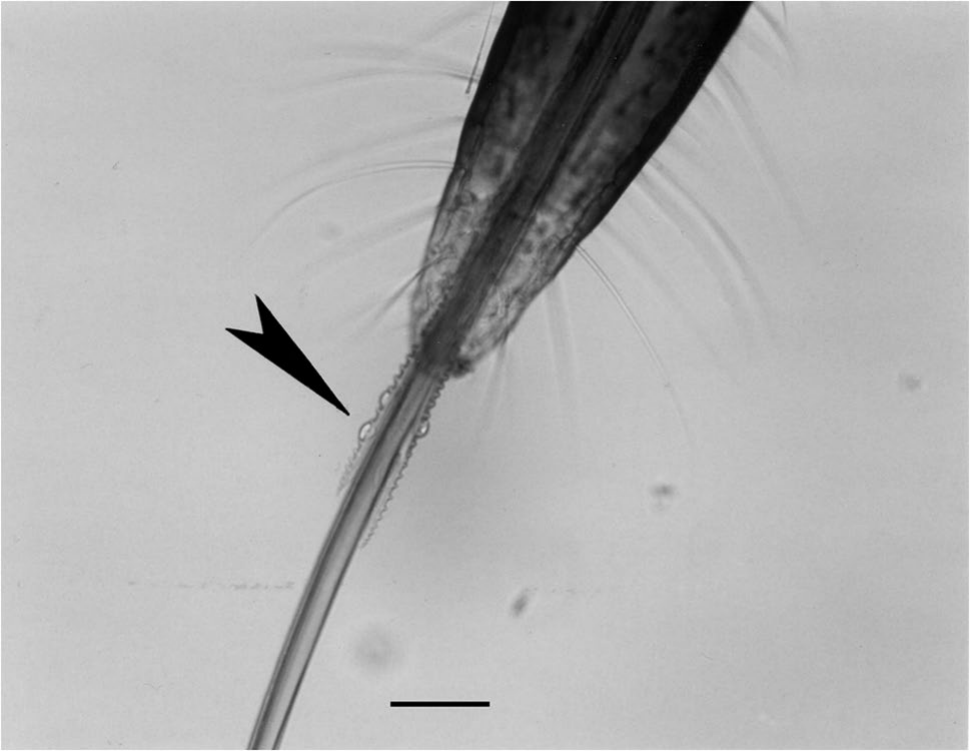
Mouthparts of a fourth instar nymph of *D. maxima* with mandibles (arrowhead) and maxillae protruded out of the proboscis (scale bar: 100 µm)

In fifth instar nymphs of *T. infestans*, the right maxilla of the mouthparts protruding into the skin was up to 3 mm long and the left one a little bit shorter. They searched for capillaries below the 0.4–0.6-mm-thick skin of mice. The size of the mouthparts increased from instar to instar. In *T. infestans*, means and standard deviations of the maximal diameter of the mandibles inclusive teeths were 17.6 ± 1.8 µm in first instar nymphs, 20.4 ± 1.3 µm in second instar nymphs, 27.4 ± 1.4 µm in third instar nymphs, 39.3 ± 1.6 µm in fourth instar nymphs and 64.7 ± 4.6 µm in fifth instar nymphs (Fig. 1). In nymphs of *D. maxima*, the respective diameters were 37.2 ± 1.3, 44.6 ± 2.1, 63.1 ± 3.3, 86.3 ± 4.7 and 97.4 ± 6.4 µm. Comparing both species, the proboscis of *T. infestans* possessed more bristles at the distal end of the proboscis (Fig. S1).

Infection rates varied according to the dose of metacyclic trypomastigotes used for the intradermal infection (Table 1). In all series, the injection of 50 to 10,000 parasites/mouse was sufficient to infect all mice. Using 10 parasites, in 2 out of 4 mice in series 1, 1 out of 6 in series 3 and 4 out of 5 mice in series 4, no parasites were detected. In xenodiagnosis of mice without detectable blood trypomastigotes, no parasites developed in any of the triatomines, indicating that there had been no latent infection. Comparing infection rates in immunodeficient mice (series 1–3) and immunocompetent mice (series 4), the variation between the different series of infections was slight using high doses, indicating that infection rates were not affected by the strain of mice, but in immunocompetent mice parasites developed in less mice after injection of 10 parasites/mouse. In those groups in which 10,000 parasites had been placed onto the feeding wound, parasites could be found in all mice of series 2, 3 and 4 but in only 3 out of 5 mice of series 1 (Table 1). According to the infection rates, 50–100 parasites had invaded the mice via the wound by the mouthparts of the triatomines.

**Table 1:** Data of infections after intradermal injection of different doses of vector-derived metacyclic trypomastigotes of *T*. *cruzi* or a placement of 10,000 of them onto the feeding wound of fifth instar nymphs of *T. infestans* or fourth instar nymphs of *D. maxima*. *n*, number of infected/uninfected mice; *pp*, prepatent periods (mean ± standard deviation of days post infection); *bt*, range of highest number of blood trypomastigotes/ml blood (× 10^6^); *ps*, period of survival (mean ± standard deviation of days post infection)

Route and dose of infection	Data	Series 1 Balb/C nu/nu	Series 2 Balb/C nu/nu	Series 3 Balb/C nu/nu	Series 4 C57 Bl/ 6
Intradermal					
10,000	*n* *pp* *bt* *ps*			4/0 15.7 ± 1.3 11–400 19.8 ± 1.3	5/0^#^ 21.8 ± 1.6 0.6–9 40.0 ± 2.6
1,000	*n* *pp* *bt* *ps*	4/0 15.8 ± 2.1 5–90 23.3 ± 2.1		4/0 17.5 ± 0.6 50–70 21.3 ± 1.0	4/0 22.5 ± 1.7 6–7 37.5 ± 1.7
100	*n* *pp* *bt* *ps*	4/0 21.3 ± 1.3 6–90 29.8 ± 4.5		5/0 19.2 ± 1.1 1–13 23.6 ± 0.5	4/0 25.7 ± 2.4 2–10 46.8 ± 6.2
50	*n* *pp* *bt* *ps*	4/0 20.5 ± 0.6 3–110 26.3 ± 0.5	4/0 21.0 ± 0.8 40–90 26.8 ± 1.3		
10	*n* *pp* *bt* *ps*	2/2 19.5 ± 0.7 90, 500 26.5 ± 0.7		5/1 22.4 ± 3.0 8–500 27.6 ± 2.8 (26.5 ± 0.9)*	1/4 29 9 53
10,000 onto wound by:		*T. infestans*	*T. infestans*	*D. maxima*	*D. maxima*
	*n* *pp* *bt* *ps*	3/2^#^ 20.7 ± 1.2 0.01, 10 28.0 ± 0.0	5/0 21.8 ± 1.6 0.04–600 26.6 ± 3.5	5/0 18.4 ± 1.5 50–200 24.6 ± 0.6	6/0 24.6 ± 2.5 2–9 46.7 ± 2.3

^#^1 infected mouse survived and is not considered in the calculation of the period of survival; *period of survival without the long living mouse

The first parasites were detected about 5 days earlier in nude mice than in immunocompetent C57 Bl/6-mice. The mean prepatent periods increased with decreasing infection doses in series 3 and 4 (Table 1). This increase was only evident in series 1 after injection of 1,000 and 100 parasites. In series 3 and 4, the mean prepatent period was correlated to the log10 of the dose of parasites (*y* = (− 2.18 *x* × log10 + 24.15); *r* = − 0.99; *y* = (− 2.48 *x* × log10 + 30.95); *r* = − 0.97, respectively). The variation was high if only 10 (series 3) or 100 parasites/mouse (series 4) had been injected. In pairwise statistic comparisons of prepatent periods within each series, in series 1 the data after injection of 1,000 and 100 parasites differed significantly (Student’s *t*-test; *p* < 0.01) and in series 3 the data of 10,000 versus 1,000 and of 1,000 versus 100. Using the data of those groups in which 10,000 parasites had been placed onto the feeding wound, in pairwise statistic comparisons of these prepatent periods to those obtained after injection of different numbers of parasites within each series, similarities are indicated if the differences were not statistically significant. This was evident in series 1 in the comparison to injections of 10 to 100 parasites/mouse, in series 2 versus 50 parasites, in series 3 versus 100 and 1,000 parasites and in series 4 versus 10,000 parasites/mouse. According to the regression equations, about 400 parasites invaded the skin in series 3 and 4. Thus, the statistics of most prepatent periods indicated individual infection doses of 10 to 1,000 parasites.

**Fig. 2:**
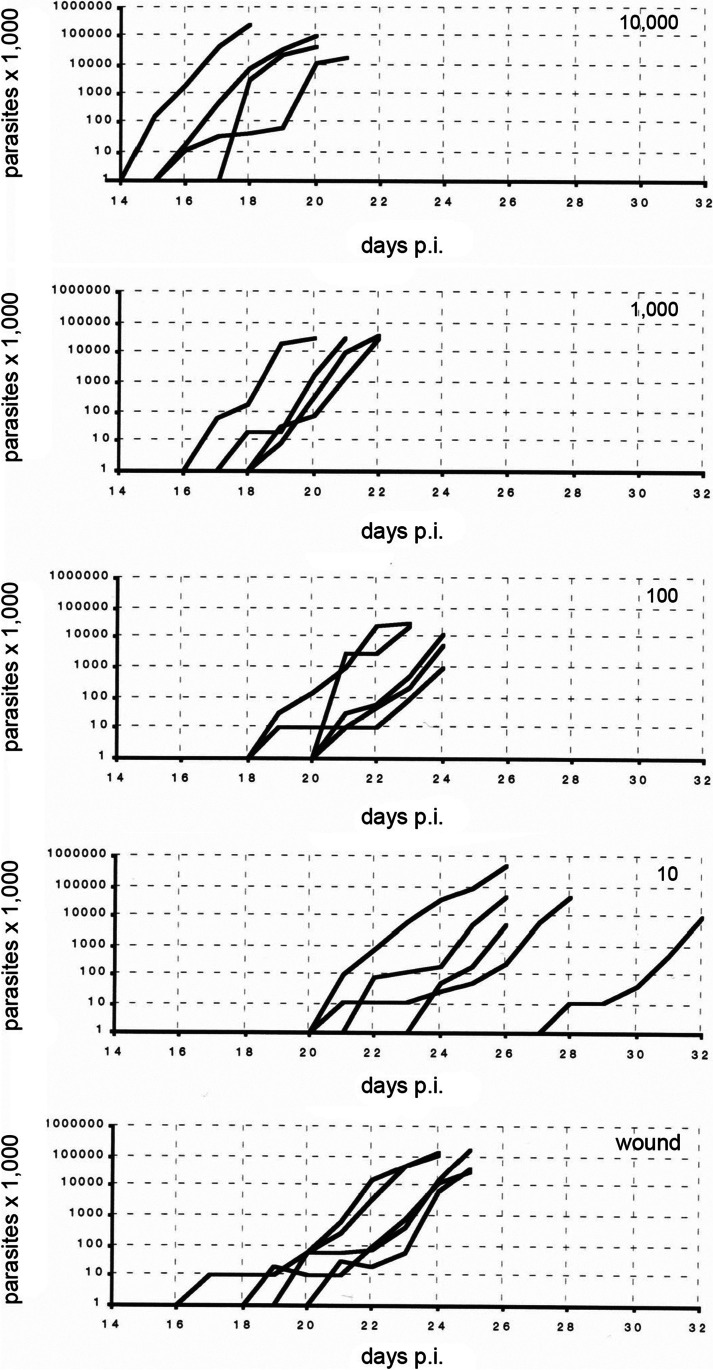
Development of *Trypanosoma cruzi* in and period of survival of immunodeficient mice after intradermal injection of 10 to 1,000 metacyclic trypomastigotes and after placement of 10,000 parasites onto the feeding wound of *Dipetalogaster maxima* (series 3)

After the prepatent period, the number of parasites initially increased exponentially. In nude mice, this continued up to 5 × 10^8^ parasites/ml blood, before they died (Fig. 2; logarithmic scale!; Figs. S2 and S3). In C57 Bl/6-mice, the number of parasites increased more slowly up to 9 × 10^8^ trypomastigotes/ml blood and was rarely lowered (Fig. S4). In both strains of mice, the parasitemias varied considerably between the individual mice of the respective group and were higher after injection of 10 trypomastigotes. Using higher doses, the individual courses of parasitemia and the maximal number of flagellates during the infection did not correlate with the injected dose. Considering the parasitemias of those groups in which 10,000 parasites had been placed onto the feeding wound, the maximal number of blood trypomastigotes in mice of groups of series 1 and 4 indicated individual infection doses of 100–1,000 parasites. In series 2 and 3, parasitemias were high, similar to those after injection of 50 and 10 metacyclic trypomastigotes, respectively.

The survival times varied strongly. Except for one mouse that survived the infection (series 4; 10,000 parasites), most infected immunodeficient mice died within 30 days and most infected immunocompetent mice within 50 days. In series 3 and 4, the mean survival time was correlated to the log10 of the dose of parasites (*y* = (− 2.57 *x* × log10 + 29.50); *r* = − 0.98; *y* = (− 4.83 *x* × log10 + 56.40); *r* = − 0.89, respectively). In pairwise statistic comparisons of the mean survival time within each series, in series 1 the data after injection of 1,000 versus 100 parasites differed significantly (Student’s *t*-test; *p* < 0.05) and in series 3 the data of 1,000 versus 100 and of 100 versus 10 (*p* < 0.01 and < 0.05, respectively). In comparisons to data of those groups with “natural infections”, no statistically significant differences were evident in series 1 in the comparisons to injections of 100 and 10 parasites/mouse (but the latter comparison considered only 4 mice), in series 2 versus 50 parasites, in series 3 versus 10 parasites and series 4 versus 100 parasites/mouse. According to the regression equations, about 100 parasites invaded the skin in series 3 and 4.

Summarising all data, infection rates indicated an invasion by 50 to 100 parasites, prepatent periods and parasitemias by 10 to 1,000 parasites, and periods of survival by 10 to 100 metacyclic trypomastigotes/mouse. Taking all data into consideration, relatively low numbers of metacyclic trypomastigotes invaded the mammalian host via the minute 65- or 86-µm-wide wound by the mouthparts of a fifth instar nymph of *T. infestans* or fourth instar nymph of *D. maxima*, respectively.

## Discussion

Vector-derived infections with *T. cruzi* are possible after drinking juices from sugarcane and guava or eating pulps of açai berries contaminated with remnants or excreta of infected triatomines and after deposition of such excreta on mucous membranes and skin wounds (Nóbrega et al. 2009; Eickhoff et al. 2013; Silva-dos-Santos et al. 2017). Placing varying numbers of metacyclic trypomastigotes onto the conjunctivae or oral mucosa of mice, a minimum of 640 and 1250 trypomastigotes, respectively, induced an infection of all mice (Kirchhoff and Hoft 1990). Placing excreta of triatomines containing 50, 500 or 5,000 metacyclic trypomastigotes inside the oropharynx or directly over a needle prick into the skin, the 50% infective dose for cutaneous challenge was 100-fold higher than that of oral challenge, indicating that oral mucosal transmission was more efficient than cutaneous transmission (Eickhoff et al. 2013).

Early investigations of the invasion of *T. cruzi* by placing infectious *T. cruzi* on the skin seemed to indicate an invasion of the intact skin (e.g. Dias 1932), but were later attributed to the previous shaving of the skin. The possibility that the feeding wound of the vector’s mouthparts offer an entry was investigated by placing 2,500 T*. cruzi* in 50 µl excreta of triatomines for 45 min onto the intact skin of a mouse and onto the feeding wound of a third instar nymph of *D. maxima* (Soares and Marsden 1986). This resulted in no infection for the intact skin and an infection rate of 24% for the feeding wound. The authors emphasised that a triatomine nearly never places its faeces directly onto its own feeding lesion and that this route of infection with *T. cruzi* must be uncommon (Soares and Marsden 1986). Placing excreta of triatomines containing 50–5,000 metacyclic trypomastigotes over the feeding wound of a triatomine, mice never became infected (Eickhoff et al. 2013). However, the developmental stage and species of the triatomines are not mentioned. According to the present measurements, the sizes of the mouthparts differ considerably, probably affecting the risk of infection, a topic of future investigations. In our system, all mice became infected after a 5- and 15-min exposure of excreta containing 15,000 metacyclic trypomastigotes onto the feeding site of fifth instar nymphs of *T. infestans* (Schuster and Schaub 2000).

In the present investigation, we placed 10,000 metacyclic trypomastigotes onto the feeding wound for 15 min and 19 out of 21 mice became infected. Therefore, parasites invade rapidly or the little feeding lesion stays open for invading parasites for some minutes. In humans, this period could be even longer, because on the contrary to mice, humans have a relatively hard and inelastic layer of ceratinocytes on the surface of the skin (Bucher and Wartenberg 1989). In addition, triatomine saliva and the histamine in a triatomines faeces cause allergic and itching reactions in humans (Harington 1958; Lumbreras et al. 1959; Costa et al. 1981; Marshall et al. 1986; Moffitt et al. 2003). Therefore, it is quite probable that faeces plus *T. cruzi* are scratched or smeared into the feeding wound if a triatomine deposits its faeces close to its feeding wound while or shortly after feeding and that parasites invade the skin by that route. The period of time between finishing blood ingestion and the deposition of faeces is the relevant factor. Many species and stages of triatomines defaecate before finishing feeding, others several minutes after having left the host (e.g. Wood 1951; Zeledón et al. 1977; Schaub and Lösch 1988; Trumper and Gorla 1991; Almeida et al. 2003; Loza-Murguía and Noireau 2010; Reisenman et al. 2011). The latter triatomines are less relevant for the direct transmission of the parasite. *T. cruzi*–infected triatomines seem to defaecate earlier after blood ingestion than uninfected specimens. However, for some *T. cruzi*–triatomine systems, such effects are not reported (reviewed by Verly et al. 2020; Guarneri and Schaub 2021; Schaub 2021).

If the number of *T. cruzi* invading via the wound of the mouthparts should be determined, a correlating response is required. Using immunocompetent mice and intraperitoneal and subcutaneous inoculations of 1, 10, 100 and 1,000 metacyclic trypomastigotes, only the period of time until the mice died was related to the dose of infection (Mshelbwala and Ormerod 1973). In addition, the number of infected mice was correlated to the infection dose, but even at the highest dose, not all mice became infected. In our system, the prepatent period and periods of time until the mice died were only correlated to the dose of parasites in series 3 (immunodeficient mice). In series 4 (immunocompetent mice), all infected mice except for one infected mouse died. Mice remaining uninfected were only present in the groups inoculated with 10 trypomastigotes (7 out of 15 mice). The prepatent period especially seemed to reflect the number of parasites infecting the mice, more evident in series 3. This was more strictly evident in infections of nude rats using 10–10,000,000 blood trypomastigotes (Schaub et al. 2001). In the present investigation, the variation of parasitemias within a group was high after receiving a low dose. This also occurred after subcutaneous infections of mice with 100 vector-derived metacyclic trypomastigotes/mouse, at least partly due to psychoneuroimmunological effects within groups of mice (Schuster and Schaub 2001a, b). The infections of the present investigation were not affected by the isolation procedure of metacyclic trypomastigotes: In 2 groups receiving parallel an intradermal injection of excreta containing 100 and 10,000 metacyclic trypomastigotes, the prepatent periods were similar to those groups receiving the isolated parasites (Heide 1999). In addition, using nymphs of *D. maxima* 1 day after resection of salivary glands of nymphs, no differences were evident to the infections via the feeding wound of normal nymphs, indicating no effect of the saliva of this species. However, in future investigations, nymphs of *T. infestans* after resection of salivary glands should be included. Effects of saliva occur in the transmission of *Leishmania* by sand flies (reviewed by Kamhavi et al. 2018).

In future investigations, the invasion via the wounds of different nymphal instars should be compared. According to the infection rates, prepatent periods and parasitemias, about 10 to 1,000 flagellates invade via the similarly sized feeding wound of fifth instar nymphs of *T. infestans* and fourth instar nymphs of *D. maxima* and these are able to cause a patent infection. The small number of parasites invading a host is important since many infection experiments were performed with high doses of *T. cruzi*, e.g. 10^5^ trypomastigotes (e.g. Ribeiro dos Santos and Hudson 1981; Mateus et al. 2019). Choosing infection doses of about 100 trypomastigotes/mouse, the variation of parasitemias within a group of mice is high but the effects reflect natural conditions.

## Supplementary Information

Below is the link to the electronic supplementary material.

Supplementary file1 (JPG 680 KB)
**Fig. S1** Mouthparts of afifth instar nymph of *T. infestans *withmandibles and maxillae protruded out of the proboscis (identical magnificationas in Fig. 1).

Supplementary file2 (DOC 785 KB)
**Fig. S2: **Development of *Trypanosoma cruzi* in and period ofsurvival of immunodeficient mice after intradermal injection of 10 to 1,000metacyclic trypomastigotes and afterplacementof 10,000 parasites onto the feeding wound of *Triatoma infestans* (series 1).

Supplementary file3 (DOC 326 KB)
**Fig. S3: **Development of *Trypanosoma cruzi* in and period ofsurvival of immunodeficient mice after intradermal injection of 50 metacyclictrypomastigotes and afterplacementof 10,000 parasites onto the feeding wound of *Triatoma infestans* (series 2).

Supplementary file4 (DOC 682 KB)
**Fig. S4: **Development of *Trypanosoma cruzi* in and period ofsurvival of immunocompetent mice after intradermal injection of 10 to 10,000metacyclic trypomastigotes and afterplacementof 10,000 parasites onto the feeding wound of *Dipetalogaster maxima *(series 4). (1 Mouse survived after injectionof 10,000 parasites).

## Data Availability

Not applicable.
