# Supplementary figures and images for: “Natural infections” with Trypanosoma cruzi via the skin of mice: size of mouthparts of vectors and numbers of invading parasites
Source: Parasitol Res. 2022 May 4;121(7):2033–41. doi: 10.1007/s00436-022-07516-5 (PMC9192721; doi:10.1007/s00436-022-07516-5)

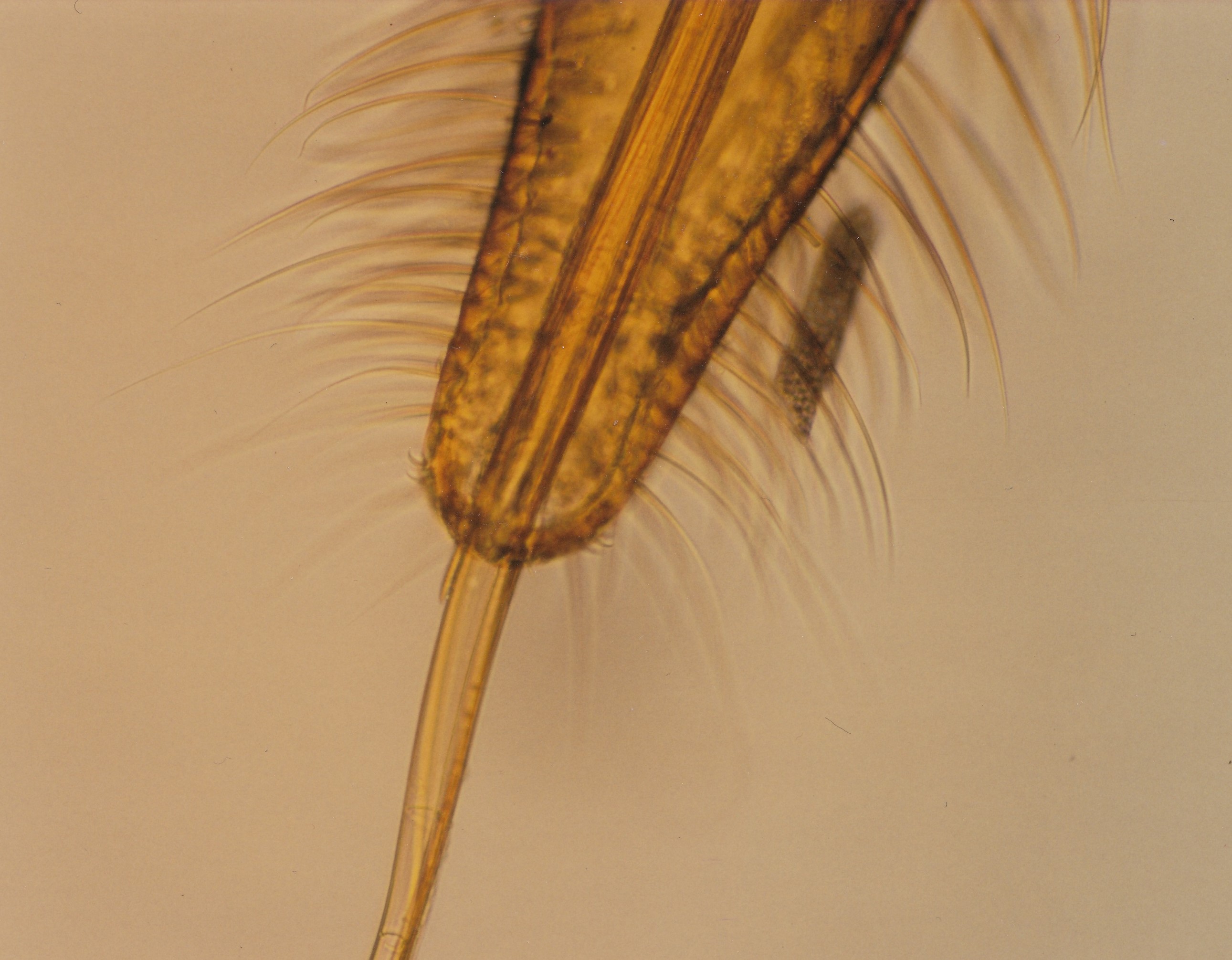

Supplement: Supplementary file 1 — Supplementary file1 (JPG 680 KB) Fig. S1 Mouthparts of afifth instar nymph of T. infestans withmandibles and maxillae protruded out of the proboscis (identical magnificationas in Fig. 1). [file 436_2022_7516_MOESM1_ESM.jpg]

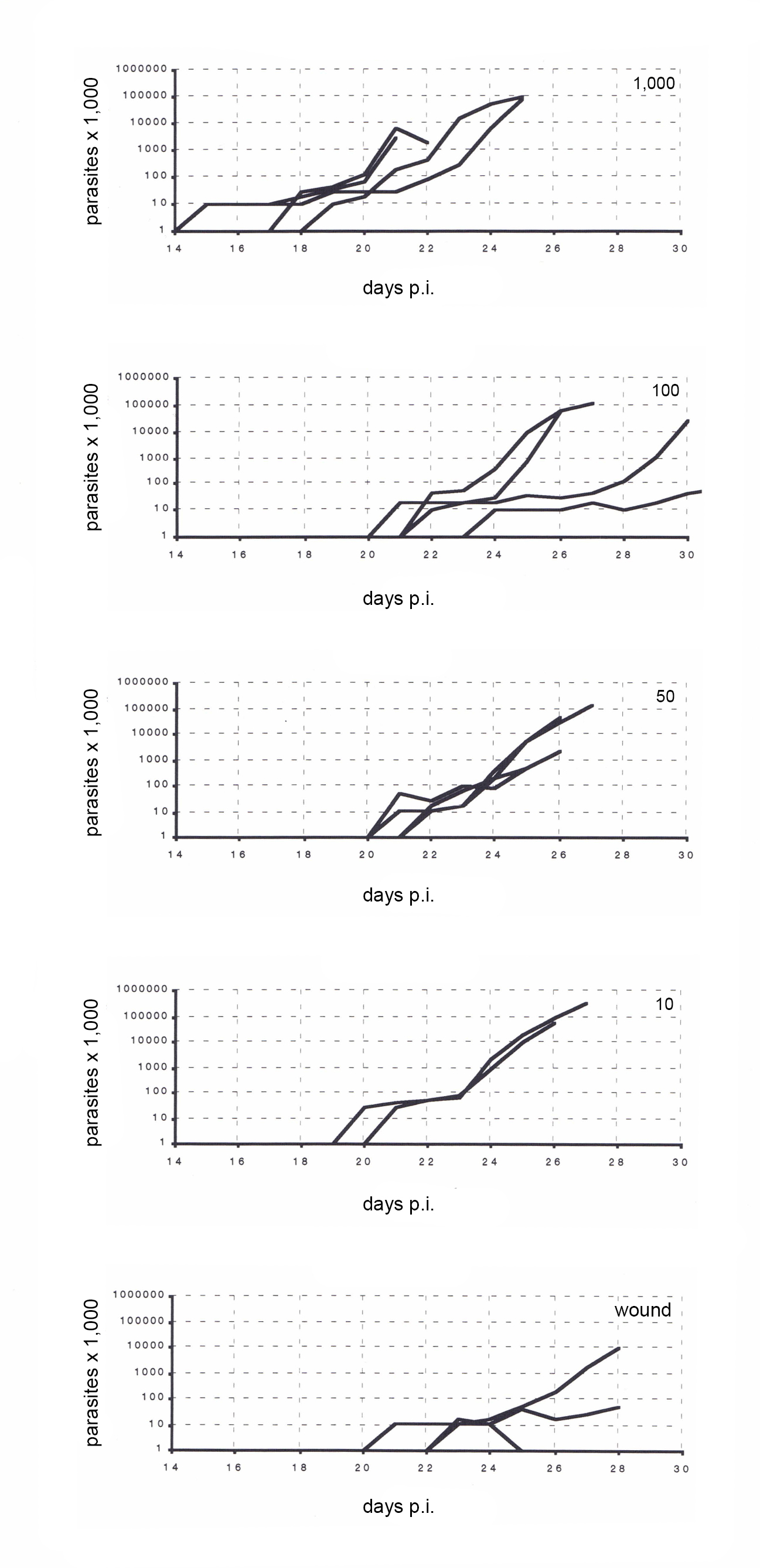

Supplement: Supplementary file 2 — Supplementary file2 (DOC 785 KB) Fig. S2: Development of Trypanosoma cruzi in and period ofsurvival of immunodeficient mice after intradermal injection of 10 to 1,000metacyclic trypomastigotes and afterplacementof 10,000 parasites onto the feeding wound of Triatoma infestans (series 1). [file 436_2022_7516_MOESM2_ESM.doc]

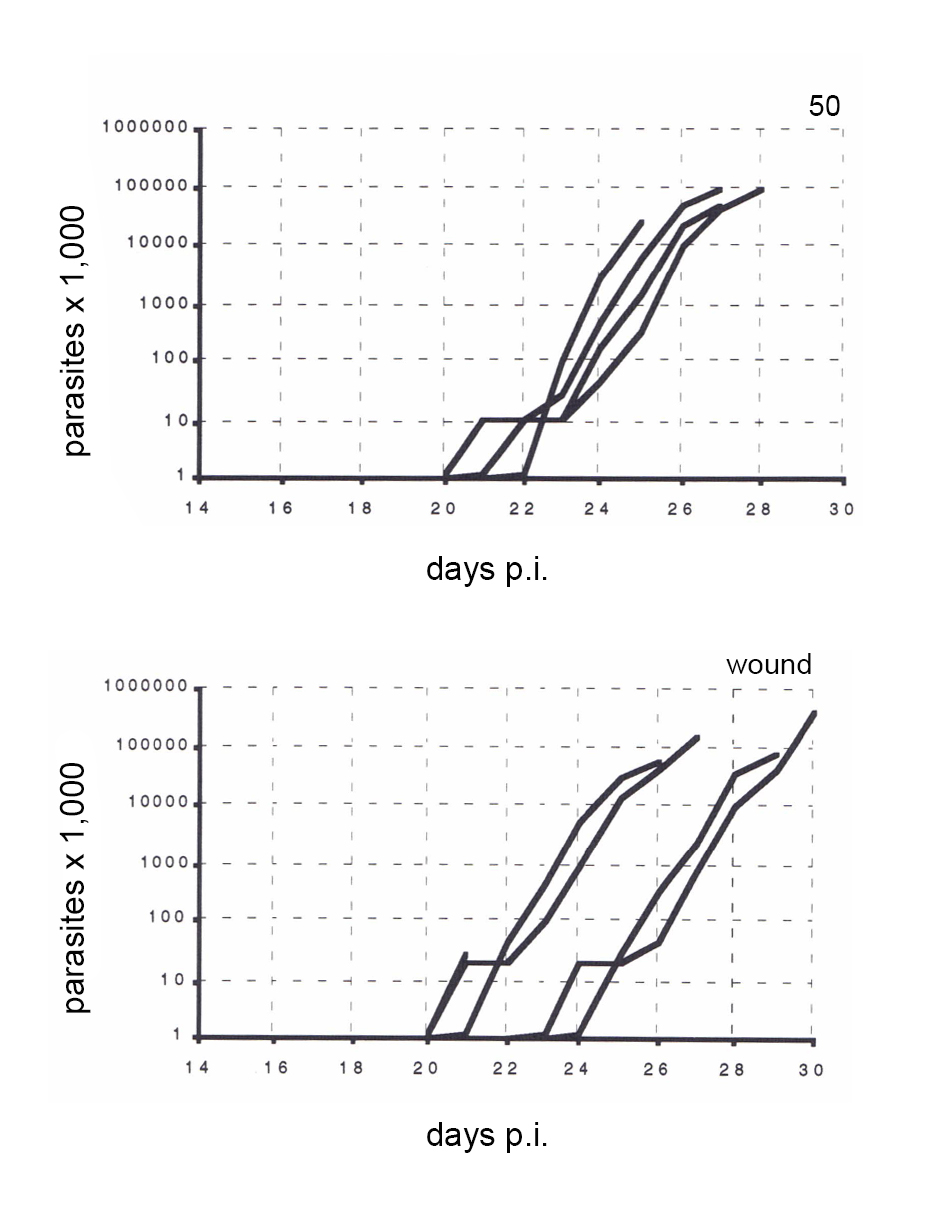

Supplement: Supplementary file 3 — Supplementary file3 (DOC 326 KB) Fig. S3: Development of Trypanosoma cruzi in and period ofsurvival of immunodeficient mice after intradermal injection of 50 metacyclictrypomastigotes and afterplacementof 10,000 parasites onto the feeding wound of Triatoma infestans (series 2). [file 436_2022_7516_MOESM3_ESM.doc]

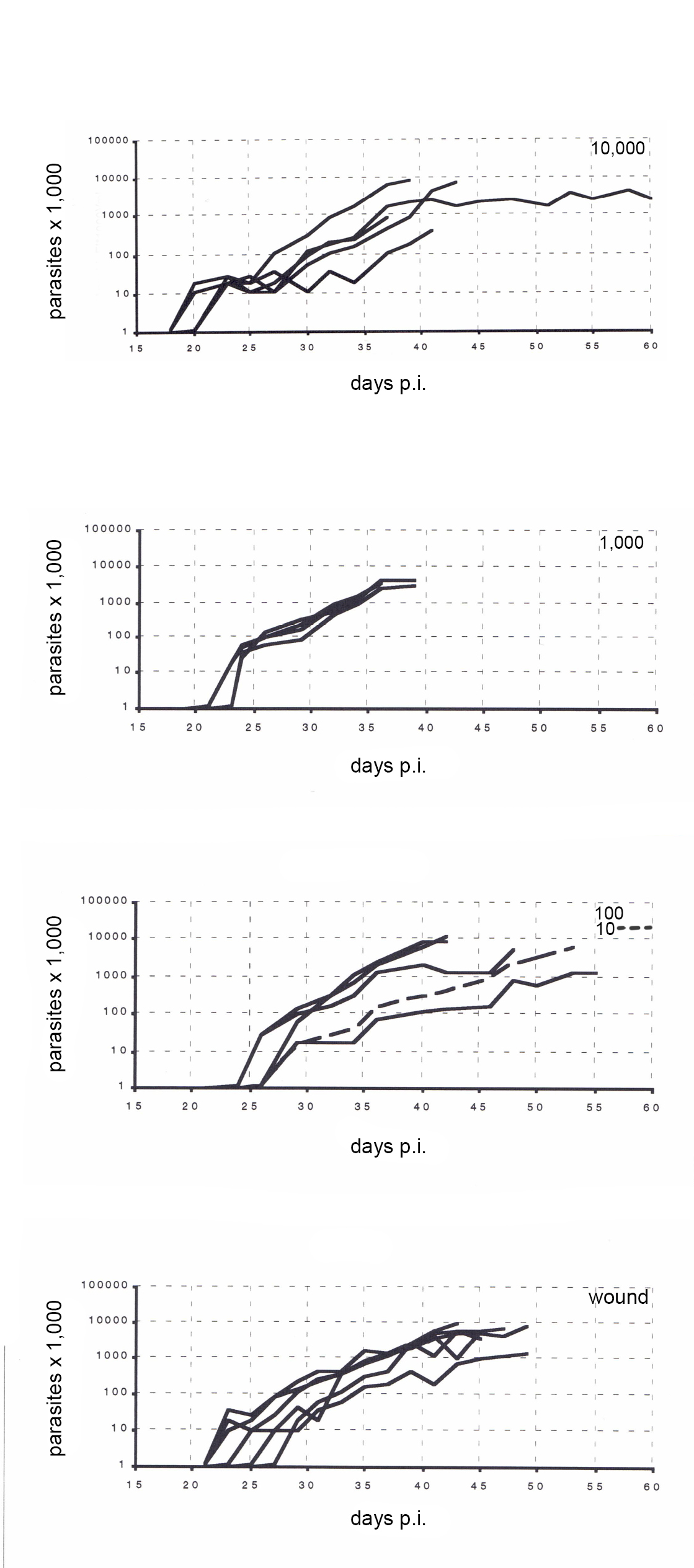

Supplement: Supplementary file 4 — Supplementary file4 (DOC 682 KB) Fig. S4: Development of Trypanosoma cruzi in and period ofsurvival of immunocompetent mice after intradermal injection of 10 to 10,000metacyclic trypomastigotes and afterplacementof 10,000 parasites onto the feeding wound of Dipetalogaster maxima (series 4). (1 Mouse survived after injectionof 10,000 parasites). [file 436_2022_7516_MOESM4_ESM.doc]
